# Optical coherence tomography characterization of spontaneous recanalized coronary thrombus – Single center experience

**DOI:** 10.34172/jcvtr.2022.30504

**Published:** 2022-11-26

**Authors:** Ankit Gupta, Raghavendra Rao K, Sreenivas Reddy S, Jeet Ram Kashyap, Vikas Kadiyala, Jaspreet Kaur, Debabrata Dash, Suraj Kumar, Munish Dev

**Affiliations:** ^1^Department of Cardiology, Government Medical College and Hospital, Sector 32, Chandigarh, 160030, India; ^2^Department of Cardiology, Post Graduate Institute of Medical Education and Research (PGIMER), Chandigarh, 160012, India; ^3^Department of Cardiology, Aster Hospitals, Mankhool, Kuwait Road, Al Mankhool, Dubai

**Keywords:** Coronary Angiogram, Optical Coherence Tomography, Percutaneous Coronary Intervention, Spontaneous Recanalized Coronary Thrombus

## Abstract

***Introduction: *** Recanalized thrombus is an under diagnosed clinical entity. Aim was to investigate the utility of optical coherence tomography (OCT) in identifying spontaneously recanalized thrombi (SRCT) for management in clinical practice.

***Methods:*** This was a retrospective study analyzing 2678 coronary angiograms over a 4-year period which included intravascular imaging guidance in 75.8% of the percutaneous coronary interventions (PCI). Angiographic suspicion of SRCT has hazy appearance seen in 34 patients.

***Results:*** Eight patients (7 males and 1 female) were confirmed with SRCT on OCT and two underwent intravascular ultrasound (IVUS). Median age was 52 years (range 33-67 years). Based on clinical symptoms, diagnosis was STEMI-2, NSTEMI-1, unstable angina-3 and chronic stable angina-2. Angiographic patterns were veiled/hazy appearances in 3; braided in 2; pseudo dissection in 2; and near occlusion in 1 patient. OCT findings displayed multiple small cavities, signal-rich with high backscattering and thin septa with smooth inner borders dividing the lumen and intercommunications. Presence of multiple holes conferred typical "Swiss cheese" or ‘lotus root’ like appearance, characteristic of recanalized thrombi. SRCT lesion length was (median interquartile ranges [IQR], 16.5[12.07-21.5] mm) and minimal luminal area (median [IQR], 1.77 [0.93-3.26] mm^2^) with significant stenosis (median [IQR], 74.0[67.0-81.0] %). Minimum/maximum number of channels were (median [IQR], 2.0[2.0-2.0]) and (median [IQR], 4.50[4.0-6.75]) respectively. Lipid rich plaque was predominant. IVUS demonstrated echo-lucent channels with small cavities. All but one patient underwent PCI.

***Conclusion:*** Intravascular imaging by OCT delineates the characteristics of recanalized thrombi and distinguishes ambiguous lesions. Majority of the lesions involving SRCT were significant both symptomatic and stenosis severity wise on OCT requiring PCI.

## Introduction

 Hazy and ambiguous lesions on coronary angiography are often a clinical dilemma for the cardiologist. Spontaneous recanalized coronary thrombus (SRCT) has been till date a pathological entity and seldom detected in clinical practice. Histopathology findings in autopsy series have characteristically identified it as multiple communicating channels separated by thin septa and the presence of a coronary lumen to be normal.^1^ The haziness on coronary angiogram can be seen in fresh thrombus, ulcerated plaque or fissure, dissection and calcification. The SRCT is a sporadic finding due to its masquerading appearance and is underdiagnosed on coronary angiography, therefore its incidence and clinical significance is unknown. Advances in intravascular imaging modalities, which include intravascular ultrasound (IVUS) and optical coherence tomography (OCT) are pivotal in overcoming the limitations of coronary angiogram. OCT in particular due to its high resolution delineates the vessel architecture and plaque features. The main purpose of this study was therefore to analyze OCT characteristics in angiographic hazy looking lesion suspicious of recanalized thrombus in aiding the diagnosis and management of SRCT in clinical practice.

## Materials and Methods

###  Study population

 This was a retrospective study which analyzed all patients from July 2017 to August 2021. A total of 2678 patients underwent invasive coronary angiography for the evaluation of coronary artery disease (CAD). The total ACS during the period were 1834 patients out of which 948 ST segment elevation myocardial infarction (STEMI), 562 non-ST segment elevation myocardial infarction (NSTEMI), 324 unstable angina (UA) and 844 stable CAD (stable angina pectoris). Of the 2186 percutaneous coronary interventions, 1658 (75.8%) were assisted by intravascular imaging (250 OCT and 1408 IVUS). Patients with angiographic suspicion of SRCT lesions were specifically looked for the OCT characteristics to confirm the diagnosis.

 ST-elevation myocardial infarction (STEMI) was considered by presence of typical angina with ST-segment elevation of 1 mm and 2 mm in two adjacent limb leads and contiguous precordial leads respectively.^2^ Non-ST elevation myocardial infarction (NSTEMI) was considered in presence electrocardiographic ST and T wave changes and elevated troponin levels. The Unstable angina (USA) had to be recent or new onset rest angina with increasing severity for more than 20 minutes duration with mild ST changes and normal troponins.^3^ Stable angina is defined as episodes of chest pain or discomfort on exertion. Clinical cardiovascular risk factors and detailed history was recorded. All patients underwent blood investigations which included renal and liver function testing along with lipid fasting lipid profiles. The exclusion criteria were the presence of cardiogenic shock, deranged liver function and elevated creatinine levels. The electrocardiograms and echocardiograms of the patients were analyzed. All the participants provided a written inform consent and Institute Ethical Committee has approved the study for the OCT data. All the procedures are in accordance with principles outlined in the Declaration of Helsinki.

###  Angiographic analysis

 Coronary angiography was performed in all patients as per standard procedure adopting appropriate views after adequate loading doses of antiplatelet therapy. The angiograms were analyzed offline by Medis Q Angio^®^ XA 7.3 (Medis medical imaging systems, Leiden, the Netherlands) the coronary angiography software for the analysis. Angiographic features associated with suspicion for SRCT included were haziness or fuzzy appearance, pseudo-dissection, braided filling defect, irregular with lobular artery outline or any combination of the above.

###  OCT imaging and analysis 

 After engaging the coronary artery with 6F/7F guiding catheter as per operator’s discretion, intravenous heparin 70-100U/Kg was administered. A guide wire was crossed across the lesion into the distal lumen. The commercially available intravascular imaging (ILUMIEN OPTIS OCT Intravascular Imaging System, St. Jude Medical, St. Paul, MN, USA) was utilized for OCT image acquisition with the help of an OCT catheter (C7 Dragonfly^TM^ OPTIS, St. Jude Medical). Using an automated pullback device at a speed of 20 mm/s, the region of interest which included distally beyond the lesion and encompassing the entire length of the lesion was scanned. A continuous flush of contrast medium into the guiding catheter enabled to create a blood free lumen for proper image acquisition. All the OCT images were recorded, only to be analyzed offline using proprietary software (St. Jude Medical) in accordance with standard recommended guidelines for the quantitative and qualitative analysis.^4^

 Quantification of the lesion severity based on OCT was calculated as the area stenosis in comparison to the largest reference area of the lesion. The lesion area stenosis of 70% was considered to be significant. The summation of the MLAs of each individual channels in the lesion at the point of tightest stenosis was considered as the minimum lumen area (MLA) of recanalized thrombus. The SRCT was defined as an OCT feature with presence of multiple channels delineated by smooth, high-luminosity with strong reflection and less attenuation in septa along with communicating channels to each other and coronary artery lumen in either end proximal and distal to the lesion.^5^ The number of channels and the channel area measurements were undertaken.

 Qualitative analysis of the plaque morphology along with plaque rupture, erosion, calcification and dissections were noted. The plaque microstructures were analyzed for cholesterol crystals and macrophages. The plaque was considered lipid rich if it was a signal poor region with ill-defined margins subtending angle of atleast 90^O^ in cross-sectional image. TCFA was considered if the fibrous cap minimum thickness of < 65μm in the presence of a lipid rich plaque. A homogeneous component with less attenuation in highly backscattered region was the fibrous plaque whereas a poor signal with distinct margins was the calcified plaque. Thin linear structures with high signal intensity were the cholesterol crystals situated in a lipid rich plaque. The signal-rich and confluent punctate regions with high intensity and reflectivity were considered as macrophages. A mass measuring at least 250μm in diameter with protrusion from the lumen surface was considered as thrombus. A cavity within a lipid rich plaque along with disruption of fibrous cap was the plaque rupture. Plaque erosion was considered in the presence of thrombus and intact plaque or luminal irregularity. Spotty calcification considered if lesions < 4 mm and subtending an arc of calcification < 90°.

 The decision for percutaneous coronary intervention was determined on the ischemia based on clinical symptoms, electrocardiogram changes and documented wall motion abnormalities on echocardiogram.

###  Statistical analysis

 The categorical data is presented as frequency and percentages (%) and Chi-square test or Fisher’s exact test was utilized as needed. Quantitative variables were represented as median with IQR (interquartile range 25^th^ – 75^th^ percentile). The statistical analysis was conducted utilizing SPSS (statistical package for the social sciences) version 28.0 (SPSS, Inc., Chicago, IL, USA).

## Results

###  Patient’s clinical characteristics 

 There were 34 angiographically detected hazy lesions suspicious of SRCT but following OCT 18 had fresh thrombus, 5 ulcerated plaque, 3 coronary arterial calcification and 8 patients had recanalized thrombus. These eight patients were identified to have characteristic features of recanalized thrombus, of which 7 were males and 1 female. Two patients underwent IVUS imaging initially reconfirmed by OCT imaging. The median age of the patients was 52 years (range 33-67 years). The clinical features of patients are listed in Table 1. Regarding the cardiovascular risk factors hypertension was observed in 4, diabetes in 2 and smoking in 2 patients. All patients had dyslipidemia. STEMI was noted in 2, NSTEMI in 1, unstable angina in 3 and chronic stable angina in 2 patients. The median left ventricular ejection fraction was 43.5% (range 35-50%).

**Table 1:** Clinical characteristics of the patients (n = 8)

**Variables**	**Patients (n=8)**
Age (years)	52.0 (37.25 - 62.75)
Male, n (%)	7 (87.5%)
Hypertension, n (%)	4 (50.0%)
Diabetes mellitus, n (%)	2 (25%)
Smoking, n (%)	2 (25%)
Family history of CAD, n (%)	0 (0%)
BMI (kg/m^2^)	26.08 (22.28 - 30.12)
BSA (m^2^)	1.80 (1.73 - 1.85)
Haemoglobin (g/dL)	12.15 (11.90 - 12.95)
Creatinine (mg/dL)	1.20 (1.10 - 1.20)
Ejection fraction, (%)	43.50 (38.50 - 45.00)
Diagnosis	
AWMI	1 (12.5%)
IWMI	1 (12.5%)
Unstable angina	3 (37.5%)
NSTEMI	1 (12.5%)
CSA	2 (25%)
Medications at admission	
Antiplatelets (%)	6 (75.0%)
Statins (%)	6 (75.0%)
ACE I/ARBs (%)	4 (50.0%)
Beta blockers (%)	3 (37.5%)
OHAs (%)	1 (12.5%)
Insulin (%)	1 (12.5%)

Abbreviations: CAD, coronary artery disease; BMI, body mass index; BSA, body surface area; AWMI, anterior wall myocardial infarction; IWMI, inferior wall myocardial infarction; NSTEMI, non-ST segment elevation myocardial infarction; CSA, chronic stable angina; ACE I, angiotensin-converting-enzyme inhibitors; ARB, Angiotensin Receptor Blockers; OHA, Oral Hypoglycemic Agents. Data are presented as mean ± SD, median (interquartile range) or n (%).

###  Angiographic features

**Figure 1:**
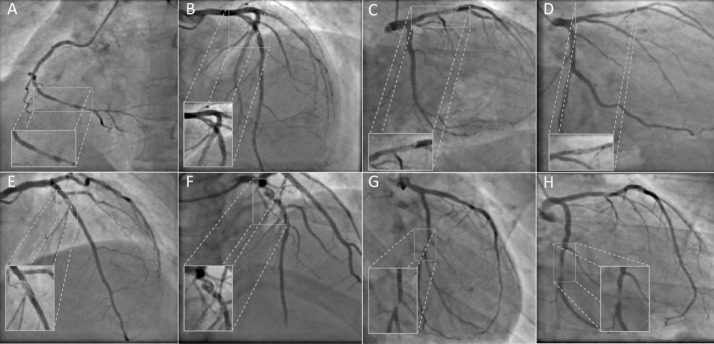
Angiographic patterns of recanalized thrombus. Panel (A-H) images corresponds to the patient #1-8. Veiled appearance (A, C and H). Pseudo-dissection (B and D). Braided like (E and F). Near occlusion (G).

 The angiographic and quantitative coronary angiography (QCA) features are listed in the Table 2. The left anterior descending artery was commonly involved in five patients. Two patients had another significant ( > 50% stenosis) lesion in other artery. Patient # 8 had recanalized thrombus in the non-culprit vessel (left circumflex artery). The angiographic lesion pattern was braided (2 patients), pseudo dissection (2 patients), veiled/hazy (3 patients) and near occlusion (1 patient) as shown in Figure 1. Mild degree of stenosis of ( < 50% diameter stenosis) was seen in 3 patients. All patients had TIMI 3 flow except one who had a TIMI 2 flow.

**Table 2:** Angiographic and QCA characteristics and procedure findings (n = 8)

**Variables**	**Patients (n=8)**
**Culprit vessel, n (%)**	
LAD	5 (62.5%)
LCX	2 (25.0%)
RCA	1 (12.5%)
**Diseased vessels, n (%)**	
SVD	6 (75.0%)
DVD	2 (25.0%)
TVD	0 (0.0%)
**ACC/AHA Lesion type, n (%)**	
Type A	5 (62.5%)
Type B_1_	1 (12.5%)
Type B_2_	2 (25.0%)
Type C	0 (0.0%)
**Lesion location, n (%)**	
Ostial	0 (0%)
Proximal	4 (50%)
Mid	2 (25%)
Distal	2 (25%)
**Baseline TIMI flow grade, n (%)**	
0	0 (0%)
1	0 (0%)
2	1 (12.5%)
3	7 (87.5%)
Haziness or filling defect, n (%)	8 (100%)
Collateral flow grade (Rentrop), n (%)	0 (0.0%)
Visible thrombus, n (%)	0 (0.0%)
Plaque rupture, n (%)	0 (0.0%)
Calcification, n (%)	0 (0.0%)
**Angiographic appearance of lesions, n (%)**	
Braided (B)	2 (25.0%)
Pseudo-dissection (P)	2 (25.0%)
Veiled/Hazy (V)	3 (37.5%)
Near occlusion (O)	1 (12.5%)
**Quantitative coronary angiography data**	
Lesion length (mm)	13.0 (12.1 - 15.5)
Diameter stenosis (%)	58.2 (41.7 - 78.6)
Reference diameter (mm)	2.67 (2.5 - 3.1)
Obstruction diameter (mm)	1.17 (0.71 - 1.60)
Area stenosis (%)	81.6 (65.9 - 95.3)

Abbreviations: QCA, quantitative coronary angiography; LAD, left anterior descending coronary artery; LCX, left circumflex coronary artery; RCA, right coronary artery; TIMI, Thrombolysis in myocardial infarction; IQR, interquartile range; SVD, single vessel disease; DVD, double vessel disease; TVD, triple vessel disease. Data are presented as median (interquartile range) or n (%).

###  OCT characteristics 

**Figure 2:**
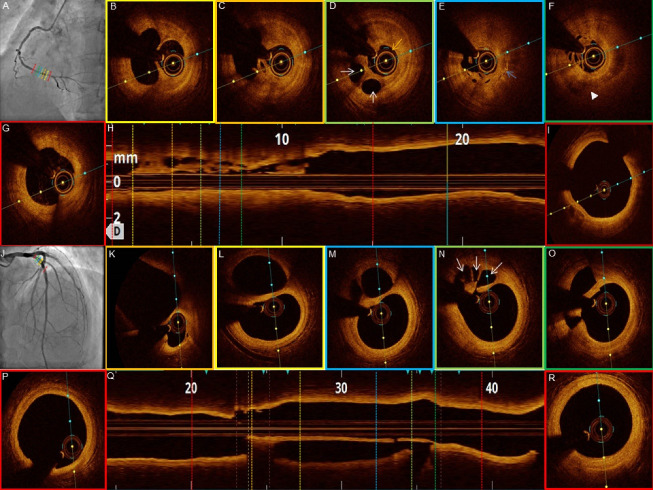
OCT images of patient #1-2 (RCA and LAD). Veiled appearance and pseudo dissection angiographic images respectively (A and J). (D) Small cavities (white arrows) and catheter fiber (yellow arrow). (N) Small cavities (white arrows). (E) Cholesterol crystals (blue arrow). (F) Lipid rich plaque (white arrowhead).

**Figure 3:**
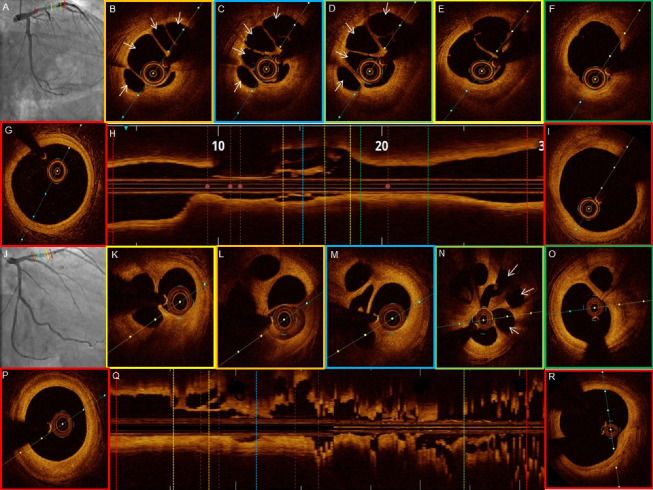
OCT images of patient #3-4 (LAD) with veiled appearance and pseudo-dissection respectively (A and J).

**Figure 4:**
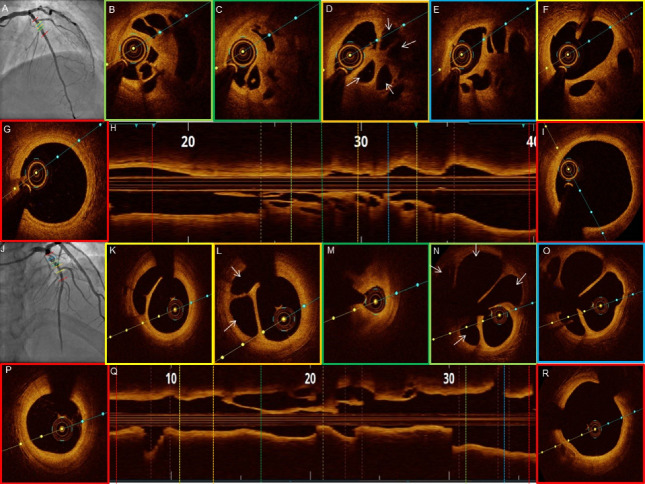
OCT images of patient #5-6 (LAD). Braided like appearance on angiogram (A and J). (D) multiple small cavities with thin septae (white arrows) giving ‘swiss cheese’ appearance. (L and N) small cavities with thin septa (‘lotus root’ or ‘swiss cheese’ appearance).

**Figure 5:**
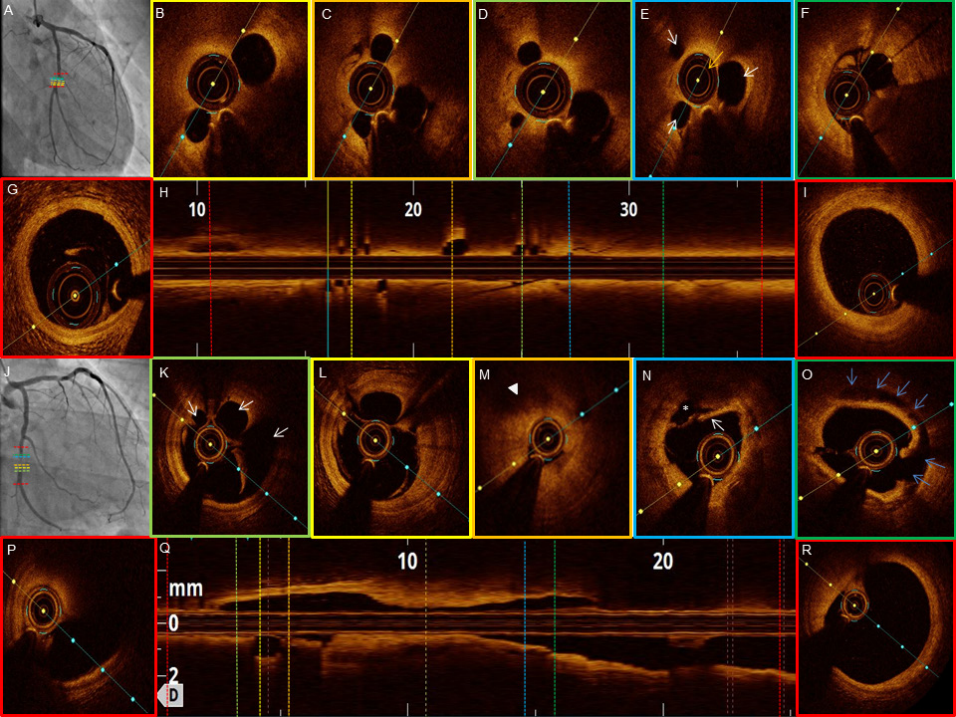
OCT images of patient #7-8 (LCX) with near occlusion and veiled appearance respectively on angiogram (A and J). (E) Small cavities (white arrows) and catheter fiber (yellow arrow). (M) Lipid rich plaque (white arrowhead). (N) Plaque rupture with cavity (white asterisk), thin cap fibroatheroma 50µ (white arrow). (O) Medial dissection (blue arrows).

 The OCT features of recanalized coronary thrombus and the plaque characteristics are listed in Table 3. All patients on OCT showed recanalized thrombi which was signal-rich with high backscattering and septa separating the lumen into multiple small cavities with inter communications. The septa were thin and inner borders had smooth lining. The presence multiple holes conferred a typical “Swiss cheese” or “lotus root” like appearance or “honeycomb” like structure. The distant areas from the fibre exhibited a weaker signal with lower luminosity (Figures 2-5). The median lesion length was 22.5 mm (IQR, 17.25-32.25 mm). The minimal luminal area was 1.71 mm^2^ (IQR, 0.93-3.26 mm^2^). The minimum channel count was 2 and the maximum number of channels varied from 4-8 with a median of 4.5 channels (IQR 4 - 6.75). The maximum channel area median was 3.08 mm^2^ (IQR, 2.09-5.19 mm^2^) and the minimum channel area noted was 0.43 mm^2^ (IQR, 0.17-0.75 mm^2^).

**Table 3:** OCT Characteristics of recanalized coronary thrombus (n = 8)

**Variables **	**Median (IQR)**
Recanalized Lesion Length (mm)	16.5 (12.07 - 21.5)
Lesion Length (mm)	22.50 (17.25 - 32.25)
Mean Reference Area (mm^2^)	8.54 (6.14 - 10.43)
Minimum Lumen Area SRCT (mm^2^)	1.7 (0.93 - 3.26)
MLA of Vessel (mm^2^)	5.88 (4.17 - 7.24)
Maximum Channel Area (mm^2^)	3.08 (2.09 - 5.19)
Minimum Channel area (mm^2^)	0.43 (0.17 - 0.75)
Vessel area at maximum channel smooth septae with high luminosity (mm^2^)	7.02 (5.86 - 8.77)
Occlusive fiber (%)	6 (75%)
Stenosis (%)	74.0 (67.0 - 81.0)
Minimum number of Channels	2.0 (2.0 - 2.0)
Maximum number of Channels	4.50 (4.0 - 6.75)
Min area/Max channel area (mm^2^)	0.29 (0.07 - 0.56)
**Plaque characteristics and mechanism of ACS **	
Spotty calcification	3 (37.5%)
Macrophages	4 (50%)
Cholesterol Crystals	3 (37.5%)
Lipid rich plaque	7 (87.5%)
TCFA	1 (12.5%)
Plaque Rupture	1 (12.5%)
Plaque Erosion	0 (0%)

Abbreviations:OCT, optical coherence tomography; SD, standard deviation; IQR, interquartile range; SRCT, spontaneously recanalized coronary thrombus; MLA, minimum lumen area; ACS, acute coronary syndrome; TCFA, thin cap fibroatheroma. Data are presented as median (interquartile range) or n (%).

###  Treatment

 All except one underwent percutaneous coronary intervention (PCI) with drug eluting stents. One patient with maximum channel area (patient #2) opted for medical management. Following PCI dual antiplatelet therapy was continued for 12 months or as appropriate to the clinical condition. The median clinical follow up period was 14 months (IQR, 3.0-41.2 months) without any adverse events.

## Discussion

 The key findings of the present study was that a) OCT is the preferred intravascular imaging modality for the diagnosis spontaneous recanalization of coronary thrombi which are visualised as hazy lesions on coronary angiography b) most lesions detected to be recanalized thrombus were functionally significant requiring revascularisation.

 Recanalization of thrombi was noted in histopathological studies. Coronary thrombus either undergo organization or recanalization leading to formation lumens of variable sizes. These vascular channels are generally lined with endothelium supported by little amount of connective tissues and the lumen varied from small to larger sizes.^1,6-9^ A certain degree of recanalization was observed in almost a third of the patients.^8,10^ Coronary angiogram due its limitations fail to identify and differentiate lesions which cause haziness such as fresh thrombus, ulcerated plaque, dissection and calcification. IVUS identifies the plaque and the lumen along with vessel areas. Due its limitation of resolution, the tissue characterization is inaccurate to identify definite plaque features and micro channels. The OCT clearly delineates the plaque characteristics due its high resolution and aids in decision making in these ambiguous lesions on coronary angiography.

 The reported prevalence of the recanalized thrombus is 0.09-0.10% of the PCIs.^11,12^ In our study the incidence was 0.36% of all PCI procedures. It comprises 0.48% of the image guidance procedures in this study. Therefore the incidence is likely to be higher when routine use of intravascular imaging is undertaken. The clinical presentation in patients with SRCT can be varied from stable angina to acute coronary syndrome. In the French registry comprising 34 SRCTs in 33 patients, the clinical mode of presentation was ACS in 30%, stable angina in 33% and silent ischemia in 30%.^12^ Two thirds of the patients were symptomatic. Whereas in a study by Xu et al, 81% of them had acute coronary syndrome.^13^ Kang et al in a study of 6 patients had ACS in three and stable angina in two patients.^5^ Likewise in the present study also majority had ACS, in six patients and stable angina in two patients.

 Most of the recanalized thrombus are found to be hemodynamically significant both clinically and on non-invasive imaging or stress imaging. Various modalities have been adopted to assess functional significance of these lesions such as myocardial scintigraphy and physiological assessment of significance by fractional flow reserve.^5,13,14^ All our patients had clinical symptoms of angina at presentation and on OCT the lesions had significant stenosis. The treatment options for the recanalized thrombus were drug-eluting balloon, drug-eluting stents and the bio-resorbable vascular scaffolds.^5,12,13,15,16^ All of the above modalities of treatment have shown favorable outcomes at follow up. Our patients underwent drug eluting stent implantation in all undergoing revascularization and had a favorable short-term outcome.

 Regarding the plaque characteristics they were mostly lipidic in nature. The underlying mechanisms of recanalized thrombus are not known. The plausible mechanisms are cardio embolic, thrombus formation after dissection in native vessel wall and at times the pathogenetic mechanisms are unclear.^17-22^ There was evidence of plaque rupture, dissection in situ along with thin cap fibro-atheroma in one of our patients. This would probably suggest that initiation could be dissection in atherosclerotic vessel wall following injury leading to thrombus which may subsequently develop recanalized thrombus. Similar findings were observed by Spinu et al in their study.^11^ In rest of our patients the mechanisms are unclear. But it is elusive as to the time frame for occurrence in recanalization of thrombus and factors predisposing to this phenomenon. Further the onset of symptoms to detection of SRCT is variable as its can be from few days to months.^23^

## Conclusion

 Recanalized thrombus is a clinically underdiagnosed entity with majority involving LAD and clinical presentation as ACS or chronic stable angina. With the advent of OCT and increased use of intravascular imaging during coronary interventional procedures the detection of SRCT and its incidence tends to be higher. Most of the lesions related to SRCT have significant stenosis which requires percutaneous intervention and favourable short term clinical outcomes.

## Acknowledgements

 We thank the secretarial assistance of Mr. Jeevan Lal and Mrs. Deepika.

## Authors Contributions

**Conceptualization:** Sreenivas Reddy S.

**Methodology: **Ankit Gupta, Sreenivas Reddy.

**Validation: **Sreenivas Reddy S.

**Formal Analysis: **Raghavendra Rao K.

**Investigation: **Sreenivas Reddy, Jeet Ram Kashyap.

**Resources: **Vikas Kadiyala.

**Data Curation: **Jaspreet Kaur.

**Writing—Original Draft Preparation: **Ankit Gupta.

**Writing—Review and Editing: **Sreenivas Reddy S.

**Visualization:** Suraj Kumar, Munish Dev.

**Supervision: **Debabrata Dash.

**Project Administration: **Sreenivas Reddy S.

## Funding

 None.

## Ethical Approval

 The study had the approval of the Institutional Ethics Committee of the Government Medical College and Hospital with reference number GMCH/IEC/2021/598/264 with date 29.06.2021. The study, as it involved the human participants was in accordance with the ethical standards of the institutional and/or national research committee and with the 1964 Helsinki Declaration and its later amendments or comparable ethical standards. All participants provided a written informed consent prior to their inclusion in the study.

## Competing Interests

 The authors declare that they have no competing interests.
